# Enterovirus 71 infection of motor neuron-like NSC-34 cells undergoes a non-lytic exit pathway

**DOI:** 10.1038/srep36983

**Published:** 2016-11-16

**Authors:** Issac Horng Khit Too, Huimin Yeo, October Michael Sessions, Benedict Yan, Eshele Anak Libau, Josephine L. C. Howe, Ze Qin Lim, Shalini Suku-Maran, Wei-Yi Ong, Kaw Bing Chua, Boon Seng Wong, Vincent T. K. Chow, Sylvie Alonso

**Affiliations:** 1 Department of Microbiology & Immunology, Yong Loo Lin School of Medicine, National University of Singapore, 117456, Singapore; 2 Immunology Programme, Life Sciences Institute, CeLS building, 28 Medical Drive, National University of Singapore, 117456, Singapore; 3 Program in Emerging Infectious Diseases, Duke-NUS Graduate Medical School, 8 College Road, 169857, Singapore; 4 Department of Laboratory Medicine, 5 Lower Kent Ridge Road, National University Hospital, 119074, Singapore; 5 Department of Anatomy, Yong Loo Lin School of Medicine, National University of Singapore, 117456, Singapore; 6 Neurobiology and Ageing Programme, Life Sciences Institute, CeLS building, 28 Medical Drive, National University of Singapore, 117456, Singapore; 7 Temasek Life Sciences Laboratory, 5 A Engineering Drive 1, National University of Singapore, 117411, Singapore; 8 Department of Physiology, Yong Loo Lin School of Medicine, CeLS building, 28 Medical Drive, National University of Singapore, 117456, Singapore

## Abstract

Enterovirus 71 (EV71) causing Hand, Foot and Mouth Disease, is regarded as the most important neurotropic virus worldwide. EV71 is believed to replicate in muscles and infect motor neurons to reach the central nervous system (CNS). To further investigate the mechanisms involved, we have employed the motor neuron cell line NSC-34. NSC-34 cells were permissive to EV71 and virus production yields were strain-dependent with differential efficacy at the entry, replication and egress steps. Furthermore, unlike all the other cell lines previously reported, EV71-infected NSC-34 cells neither displayed cytopathic effect nor underwent apoptosis. Instead, autophagy was markedly up-regulated and virus-containing autophagic vacuoles were isolated from the culture supernatant, providing the first experimental evidence that EV71 can adopt a non-lytic exit pathway. Finally, the ability of EV71 to infect productively NSC-34 cells correlated with its ability to invade the CNS *in vivo*, supporting the relevance of NSC-34 cells to study the intrinsic neurovirulence of EV71 strains.

Following the success of WHO Global Poliovirus Eradication Programme, Enterovirus 71 (EV71) is now regarded as the most important neurotropic virus in the world^1^. EV71 is a non-enveloped, single-stranded, positive sense RNA virus from the family *Picornaviridae*. It causes hand, foot and mouth disease (HFMD) mainly in young children below 4 years of age that usually presents itself as a self-limiting mild febrile disease with symptoms including ulcers in the mouth, maculopapular rashes, or blister-like eruptions on the palms and soles^2^. However, severe central nervous system (CNS) complications have been associated with EV71 infections, such as brainstem encephalitis, aseptic meningitis, pulmonary edema and cardiopulmonary collapse^3^,^4^,^5^. In addition, patients who recover from severe disease may develop long term neurologic and psychiatric disorders^6^,^7^,^8^. Outbreaks have been reported throughout the world including Singapore, Malaysia, Vietnam, Taiwan, Cambodia, China, Australia and Japan^9^,^10^,^11^,^12^,^13^,^14^,^15^,^16^. Currently, there are no effective prophylactic or therapeutic agents against EV71 although recent progress has been made^17^.

Unlike poliovirus, the neuropathogenesis of EV71 is still not well understood. EV71 has been proposed to reach the CNS via retrograde axonal transport, whereby the virus actively replicates in skeletal muscles, infects motor neurons at the neuro-muscular junctions and enters the CNS to eventually accumulate in the brainstem^3^,^18^,^19^,^20^. *In vitro*, EV71 neurovirulence has been studied in several neuronal cell lines including SK-N-SH (neuroblastoma)^21^, SH-SY5Y (neuroblastoma)^22^, SF268 (glioblastoma)^23^, RBA-1 (astrocyte)^24^, and U251 (astrocyte)^25^. However, as neuronal cells isolated from different tissues display different features and characteristics, it is possible that data obtained with these cell lines may not reflect accurately the events occurring at the neuromuscular junctions during infection. Here, we report the use of the motor neuron cell line NSC-34 to study EV71 neurovirulence. NSC-34 is a hybrid cell line obtained from the fusion between mouse neuroblastoma and motor neuron-enriched embryonic mouse spinal cord cells^26^. NSC-34 cells display motor neuron-like properties, including the ability to generate action potentials and to produce, store and release acetylcholine^26^. NSC-34 cells have been employed to study the pathogenesis of motor neuron degenerative diseases such as amyotrophic lateral sclerosis^27^,^28^,^29^,^30^,^31^. Besides motor neurons in the ventral horn of the spinal cord, motor neurons in the trigeminal motor nucleus, facial motor nucleus, nucleus ambiguus and hypoglossal nucleus could also represent points of entry for EV71 to the brainstem from oropharyngeal lesions. Therefore, investigating the ability of EV71 to infect motor neuron-like cells is highly relevant and likely to provide insights into the mechanisms employed by EV71 to invade the CNS.

## Results

### The NSC-34 motor neuron cell line is permissive to EV71 infection

**Figure 1:**
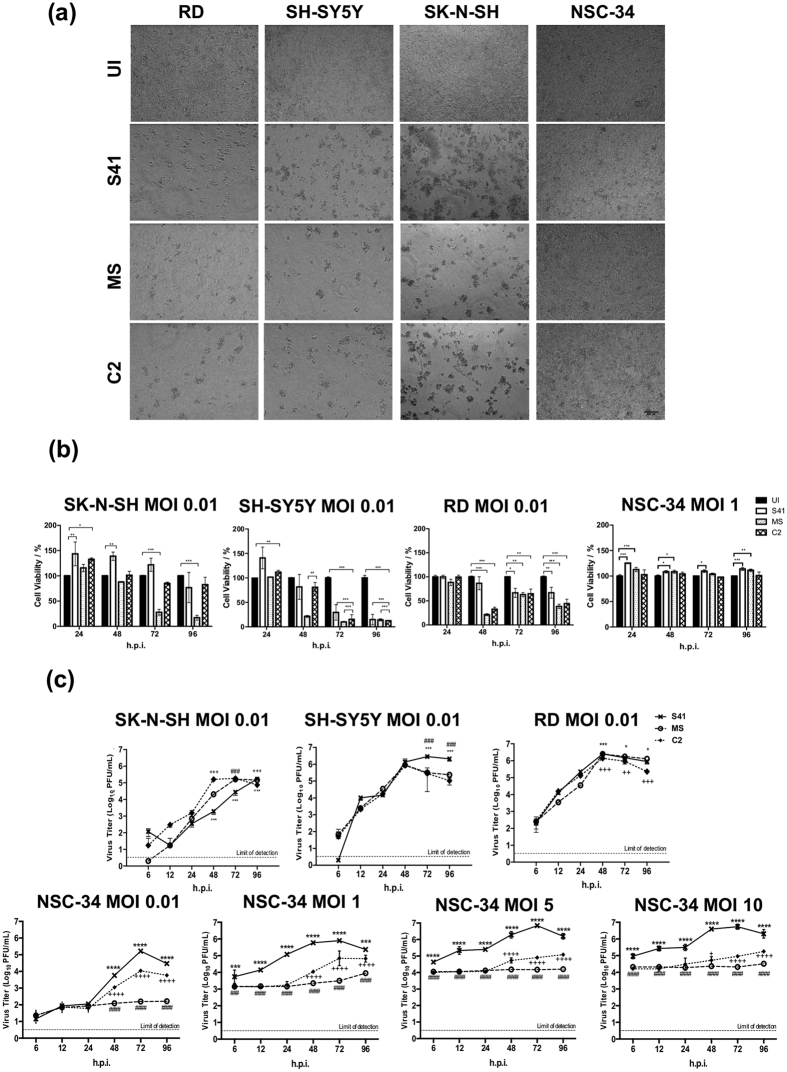
Morphological changes, viability and virus titers of EV71-infected cultures. (**a**) Phase-contrast microscopic images of EV71-infected RD, SH-SY5Y, SK-N-SH and NSC-34 cells (MOI 1) at 96 h.p.i. The images were captured at 20× magnification. Representative views are shown. UI, uninfected control. (**b**) Cell viability of EV71-infected RD, SK-N-SH, SH-SY5Y and NSC-34 cells was determined using alamarBlue™ viability assay. Data are expressed as the mean ± SD of technical triplicates. Statistical analysis was performed using two-way ANOVA test with Bonferroni correction (^*^p < 0.1, ^**^p < 0.01, ^***^p < 0.001). (**c**) SK-N-SH, SH-SY5Y, RD and NSC-34 cells were infected with S41, MS and C2 strains at the indicated MOIs. At the indicated time points post-infection, the virus titers in the culture supernatants were determined by plaque assay. The data are expressed as the mean ± SD of technical triplicates. Statistical analysis was performed using two-way ANOVA test with Bonferroni correction. Legends: ^*^statistical analysis between S41- and C2-infected cells; ^#^statistical analysis between S41- and MS-infected cells; ^+^statistical analysis between MS- and C2-infected cells. ^*,#^ or ^+^*p* < 0.1, ^**, ##^ or ^++^*p* < 0.01, ^***, ###^ or ^+++^*p* < 0.001. These experiments were performed twice independently. One representative set is shown. Scale bar denotes 100 μm.

NSC-34 cells were infected with three different EV71 clinical isolates namely S41, MS and C2 strains (Table S1), and cell morphology changes were microscopically monitored and compared to human rhabdomyosarcoma (RD), and neuroblastoma SH-SY5Y and SK-N-SH cell lines. As previously reported^21^,^32^,^33^, RD, SH-SY5Y and SK-N-SH cells displayed typical cytopathic effect (CPE) characterized by cells rounding up and eventually detaching from the bottom of the well (Fig. 1a) by 96 hours post-infection (h.p.i.). In contrast, EV71-infected NSC-34 cells remained intact with no evidence of CPE and continued to grow over time (Fig. 1a). Cell viability assay indicated that NSC-34 cells infected with EV71 at MOI 1 remained viable over a 96 hour-time course unlike RD, SH-SY5Y and SK-N-SH cells which displayed rapid and gradual loss in viability over time at MOI as low as 0.01 (Fig. 1b).

Presence of infectious virus particles in the culture supernatant from EV71-infected NSC-34 cells was readily detected, indicating that the lack of CPE and cell death observed is not due to the inability of the virus to enter and/or replicate effectively in these cells (Fig. 1c). Infection of NSC-34 cells (MOI 0.01) resulted in overall lower virus titers as compared to RD and neuroblastoma SH-SY5Y and SK-N-SH cells (Fig. 1c). Infection of NSC-34 cells with S41 strain gave rise to higher virus titers than those obtained with C2 and MS strains at all the MOIs tested with a peak at 72 h.p.i. (Fig. 1c). In contrast, no substantial increase in virus titer was observed with MS strain over the 96 hour-time course which indicates a limited productive infection. Interestingly, the respective ability of each virus strain to infect productively RD, SK-N-SH, and SH-SY5Y did not mirror the infectivity trends observed in NSC-34 cells. This observation suggests that EV71 infection profile is strain and cell line-dependent, and care should be taken in data interpretation and extrapolation to the neurovirulence potential of EV71 strains.

### EV71 replicates actively in NSC-34 cells

**Figure 2:**
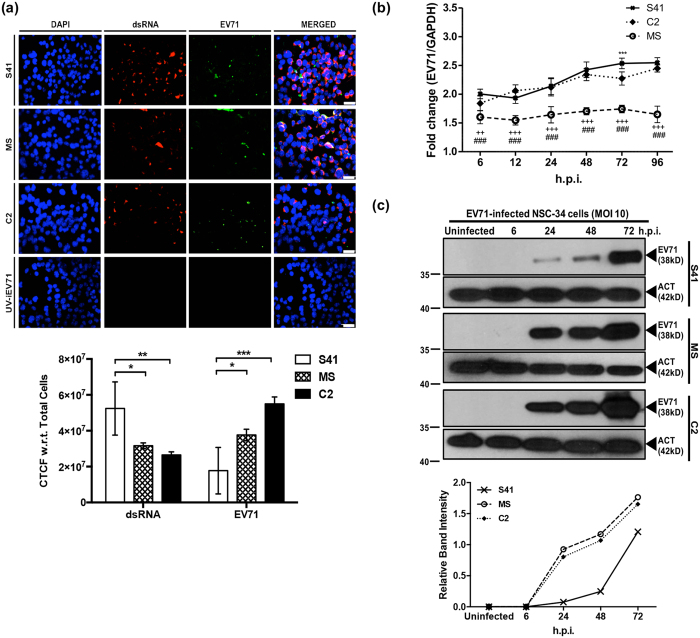
Intracellular viral genome replication and viral protein translation in NSC-34 cells. (**a**) IF assay. NSC-34 cells were infected with S41, C2 and MS strains at MOI 10, or with UV-inactivated EV71 (UV-iEV71). At 72 h.p.i., the infected cells were fixed with ice-cold methanol and processed for immunostaining using antibodies specific to dsRNA (red) and VP0/VP1 capsid protein (green). The images were captured at 40× magnification. Scale bar denotes 30 μm. Representative views are shown. The corrected total cell fluorescence (CTCF) was measured using ImageJ according to the equation: CTCF = Integrated Density – (Area of selected cell × Mean fluorescence of background readings). Statistical analysis was performed using two-way ANOVA with Tukey’s multiple comparisons test (^*^p < 0.1, ^**^p < 0.01, ^***^p < 0.001). Data are expressed as the mean ± SD of three different microscopic views. (**b**) Quantitative real-time PCR. NSC-34 cells were infected with S41, C2 and MS strains at MOI 1. At various time points post-infection, the cells were harvested and intracellular viral RNA was extracted for real time PCR analysis using VP1 primers specific to each virus strain. The viral RNA titers were normalized to GAPDH RNA levels in each sample and expressed as fold changes (EV71 expression/GAPDH expression). The data are expressed as the mean ± SD of technical triplicates. Legends: ^*^statistical analysis between S41- and C2-infected cells; ^#^statistical analysis between S41- and MS-infected cells; ^+^statistical analysis between MS- and C2-infected cells. ^*,#^ or ^+^*p* < 0.1, ^**, ##^or ^++^*p* < 0.01, ^***, ###^ or ^+++^*p* < 0.001. (**c**) Western blot analysis of EV71-infected NSC-34 cell lysates using an anti-VP0/VP1 primary antibody. β-actin was used as loading control and the band intensities were analysed using ImageJ software. Gels images were cropped. All these experiments were performed twice independently. One representative set is shown.

To confirm that NSC-34 cells are permissive to EV71 and support viral replication, double stranded RNA (dsRNA), an intermediate RNA species produced during viral replication, was detected by immunofluorescence (IF). The main capsid protein VP1 and its precursor VP0 were also detected. Results showed that whereas no signal was detected with cells incubated with UV-inactivated virus (negative control), dsRNA and VP1/VP0 signals were detected with NSC-34 cells infected with each of the three EV71 strains (Fig. 2a). Interestingly, greater signal intensity for VP0/VP1 protein was measured in MS- and C2-infected cells compared to S41-infected cells, whilst greater signal intensity for dsRNA was detected for S41-infected cells compared to C2 and MS-infected cells (Fig. 2a). This observation suggests different viral protein accumulation in MS- and C2-infected cells due to different egress efficiency.

The rate of intracellular viral replication of S41, MS and C2 strains in NSC-34 cells was further examined by quantitative real-time PCR. Presence of VP1 RNA was readily detected in NSC-34 cells infected with each of the three strains over the 96-hour course of infection at MOI 1 (Fig. 2b). Since different VP1-specific primers sets were used for each virus strain, it was not possible to compare the replication efficacy between the three virus strains. However, we could observe that whereas the relative amount of viral RNA increased over time for both S41 and C2 strains, it remained constant for MS strain, mirroring the virus titer kinetics measured in the culture supernatant (Fig. 1). This latter observation thus further supports that MS strain replicates less efficiently in NSC-34 cells compared to S41 and C2 strains.

Next, intracellular production of the viral capsid protein VP0/VP1 was analysed by Western blot. A positive signal was detected at 24 h.p.i. onwards and the signal intensity increased over time post-infection for each EV71-infected cell lysate (Fig. 2c). Interestingly, greater band signal intensities were observed with C2- and MS-infected cell lysates compared to S41-infected lysate (Fig. 2c). The apparent discrepancy between intracellular viral protein production and presence of virus particles in the culture supernatant suggests that egress of mature MS and C2 virus particles from NSC-34 cells may not be as efficient as for S41 strain, thereby resulting in protein intracellular accumulation. This finding is also consistent with the IF observations noted above.

**Figure 3:**
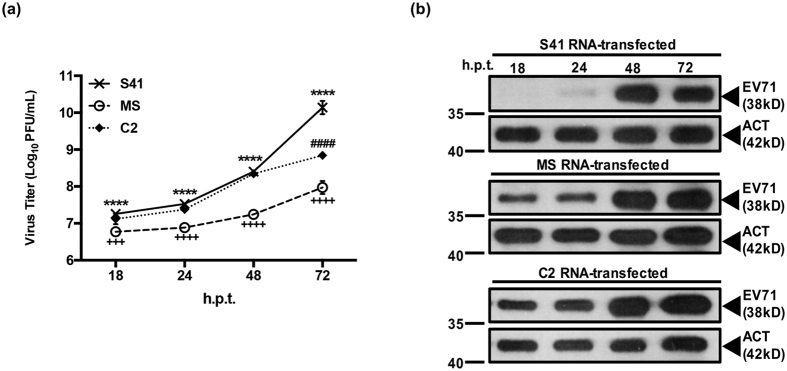
Virus production in NSC-34 cells transfected with purified virus genome. NSC-34 cells were transfected with 0.25 μg of S41, MS and C2 viral genome RNA. (**a**) At the indicated time points post-transfection, virus titers in the culture supernatants were determined by plaque assay. Data are expressed as the mean ± SD of technical triplicates. Legends: ^*^statistical analysis between S41- and C2-infected cells; ^#^statistical analysis between S41- and MS-infected cells; ^+^statistical analysis between MS- and C2-infected cells. ^*,#^ or ^+^*p* < 0.1, ^**, ##^ or ^++^*p* < 0.01, ^***, ###^ or ^+++^*p* < 0.001 (**b**) Western blot analysis of cell lysates using anti-VP0/VP1 primary antibodies. The band intensities were normalized against β-actin and analysed using ImageJ software. Gels images were cropped. These experiments were performed twice independently. One representative set is shown.

To further study the ability of EV71 to replicate in NSC-34 cells, the viral RNA genome of S41, MS and C2 strain was purified and directly transfected into NSC-34 cells. The amount of viral RNA to be transfected was optimized, and 0.25 μg was found to result in productive infection without causing cytotoxicity (Fig. S1). The kinetic profiles of virus production obtained with transfected NSC-34 cells indicated that production of MS virus particles was clearly lower compared to C2- and S41-transfected cultures throughout the course of the experiment (Fig. 3a). S41 and C2 displayed similar production yields over time with the exception of 72 h.p.i. at which time point S41 virus titer was significantly higher than C2 titer (Fig. 3a). This is in contrast with the kinetic profiles obtained upon infection with the whole virus, where virus titers measured in the culture supernatant of S41-infected NSC-34 cells (MOI 1 and above) were significantly higher than those measured in C2-infected cells at all the time points studied (Fig. 1). This observation suggests that C2 virus may be impaired at the initial entry step compared to S41 strain. In addition, Western blot analysis of the cell lysates prepared at different time points post-transfection showed greater intracellular viral protein accumulation in MS- and C2-transfected- than S41 transfected cells (Fig. 3b), which supports our hypothesis that MS and C2 strains may be less efficient than S41 at egressing from NSC-34 cells.

Altogether, the data support that NSC-34 cells are permissive to infection with non mouse-adapted EV71 strains with active replication and production of infectious virus particles that did not result in overt cytopathic effect. However, the production yield of virus particles in the culture supernatant was found to be strain-dependent with possible differences in both the entry- and egress efficacy between strains.

### Entry of S41, MS and C2 strains into NSC-34 cells is not mediated by mSCARB-2 and mPSGL-1 receptors

Prior studies have identified human SCARB-2 (hSCARB-2) and PSGL-1 (hPSGL-1) as the main receptors that mediate entry of EV71 into its human host cells^34^,^35^. To determine whether mSCARB2 mediates EV71 entry into NSC-34 cells, an *in vitro* competition assay was performed using a commercially available anti-mouse SCARB-2 (mSCARB-2) antibody. The mouse-adapted EV71:TLLm strain which was previously shown to enter mouse cells via mSCARB-2 receptor^36^, was used as a positive control. Expectedly, incubation of NSC-34 cells with mSCARB-2 antibody prior to infection with EV71:TLLm strain led to significant reduction of virus titers in the culture supernatant (Fig. S2a). In contrast, incubation of NSC-34 cells with mSCARB2 antibodies prior to infection with S41, C2 or MS strain did not affect the virus titers (Fig. S2b).

In addition to play a role in virus entry, SCARB-2 has also been reported to be essential for intracellular uncoating of EV71 virions by inducing a conformational change^34^. To further investigate the role of SCARB2 during EV71 infection in NSC-34 cells, a siRNA SCARB-2 knockdown approach was undertaken. Western blot confirmed efficient silencing of SCARB2 gene expression in siRNA-transfected NSC-34 cells (Fig. S2c&d). Interestingly, a significant dose-dependent decrease in virus titers was observed in SCARB-2 silenced NSC-34 cells (Fig. S2c). This observation thus indicates that while mSCARB-2 may not be involved in virus entry, it may play a role in virus uncoating in NSC-34 cells.

Of note, the mPSGL-1 receptor was not found to be expressed in NSC-34 cells as evidenced by Western blot analysis (data not shown), hence, the mechanism of EV71 entry into NSC-34 cells remains to be further investigated.

### EV71-infected NSC-34 cells do not undergo apoptosis

Apparent lack of CPE in EV71-infected NSC-34 cells could be due to a significantly lower infectivity of NSC-34 cells compared to RD cells thereby leading to a small percentage of infected cells whose cyptopathic phenotype may go undetected. To address this hypothesis, the infectivity of NSC-34 cells was determined over time and compared to RD cells. Briefly, NSC-34 and RD cells were infected with EV71 S41 strain at MOI 10 and 1, respectively. At 3, 6, 9, 12, 24, 48 and 72 hours post-infection, monolayers were washed thoroughly and processed for immunostaining using anti-EV71 antibodies. Results showed that the percentage of infected NSC-34 cells ranged between 50% (3 h.p.i.) and 90% (72 h.p.i.) which was comparable to infected RD cells (Fig. S3). Thus, this result indicated that the infectivity of NSC-34 at MOI 10 was comparable to that observed with RD cells infected at MOI 1. This finding thus supports that absence of CPE observed with EV71-infected NSC34 cells (MOI 10) is not due to the fact that only a minority of cells are infected. It suggests instead that exit of EV71 relies on a non-lytic mechanism in NSC-34 cells.

**Figure 4:**
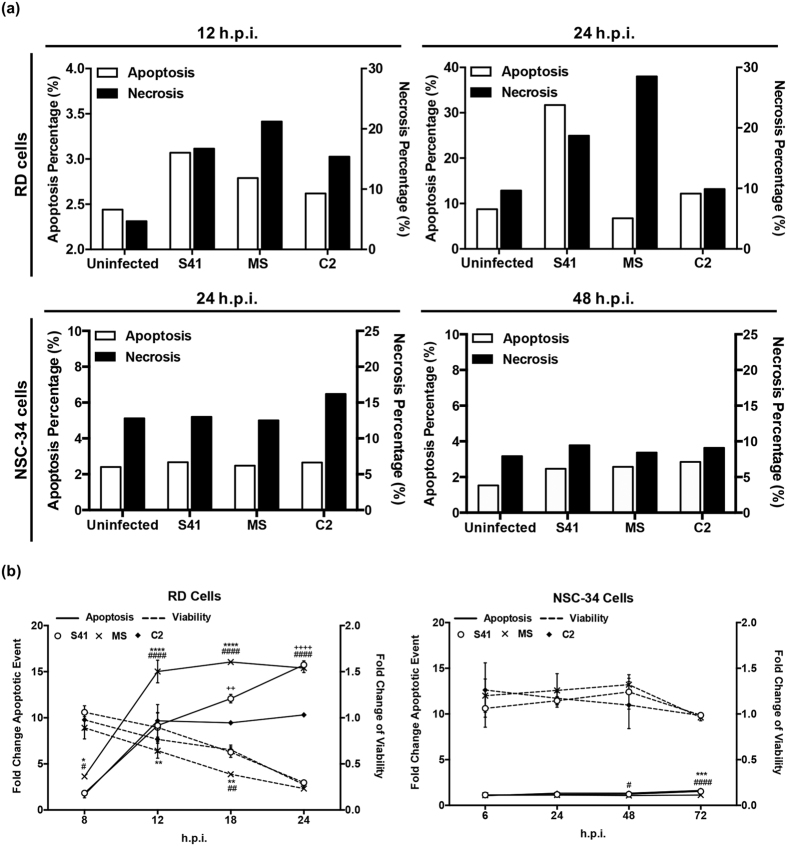
Apoptosis in EV71-infected RD and NSC-34 cells. RD and NSC-34 cells were infected with S41, C2 and MS strains at MOI 1 and 10, respectively. (**a**) Annexin V/ Propidium Iodide staining. At the indicated time points post-infection, the cells were harvested and stained for Annexin V and Propidium Iodide, prior to FACS analysis (see plots in Fig. S3). Data are expressed as the percentage of necrotic or apoptotic cells. (**b**) Cell viability and caspase activation. At the indicated time points post-infection, the cells were harvested and processed in the ApoLive-Glo™ multiplex assay. Data are expressed as the mean ± SD of technical triplicates. Statistical analysis was performed using two-way ANOVA with Tukey’s post test. Legends: ^*^statistical analysis between S41- and MS-infected cells; ^+^statistical analysis between S41- and C2-infected cells; ^#^statistical analysis between MS- and C2-infected cells. ^*,#^ or ^+^*p* < 0.05, ^**, ##^ or ^++^*p* < 0.005, ^***, ###^ or ^+++^*p* < 0.0005, ^****, ####^ or ^++++^*p* < 0.0001. These experiments were performed twice independently. One representative set is shown.

To further study the absence of both CPE and viability loss in EV71-infected NSC-34 cells, we asked whether these cells undergo apoptosis upon EV71 infection, a feature that has been previously reported for EV71-infected RD^37^,^38^, SK-N-SH^21^ and SH-5YSY^19^ cells. Using annexin-V/PI double staining, we confirmed that human muscle RD cells infected with MS, C2 or S41 strain clearly displayed apoptosis (Fig. 4a and Fig. S4), whereas murine motor-neuron derived EV71-infected NSC-34 cells did not show significant apoptosis, even though these cells showed apoptosis after treatment with a well-known apoptosis inducer, staurosporine^39^ (Fig. S4).

Since apoptosis in EV71-infected neuroblastoma SK-N-SH and SH-5YSY could not be assessed by Annexin V/PI staining due to the fragility of the cells, TUNEL assay was performed. While increased OD readings were obtained in EV71-infected SK-N-SH and SH-5YSY cells compared to uninfected control cells, no significant difference was seen between EV71-infected and uninfected NSC-34 cells (Fig. S5). These data thus support that EV71-infected NSC-34 cells do not undergo significant apoptosis.

The lack of apoptosis in EV71-infected NSC-34 cells was further confirmed in an *in vitro* assay which combines two assay chemistries assessing both viability and caspase activation events. While EV71-infected RD cells underwent apoptosis and lost viability over time (Fig. 4b), EV71-infected NSC-34 cells did not undergo significant apoptosis and remained viable throughout the experiment (Fig. 4b).

**Figure 5:**
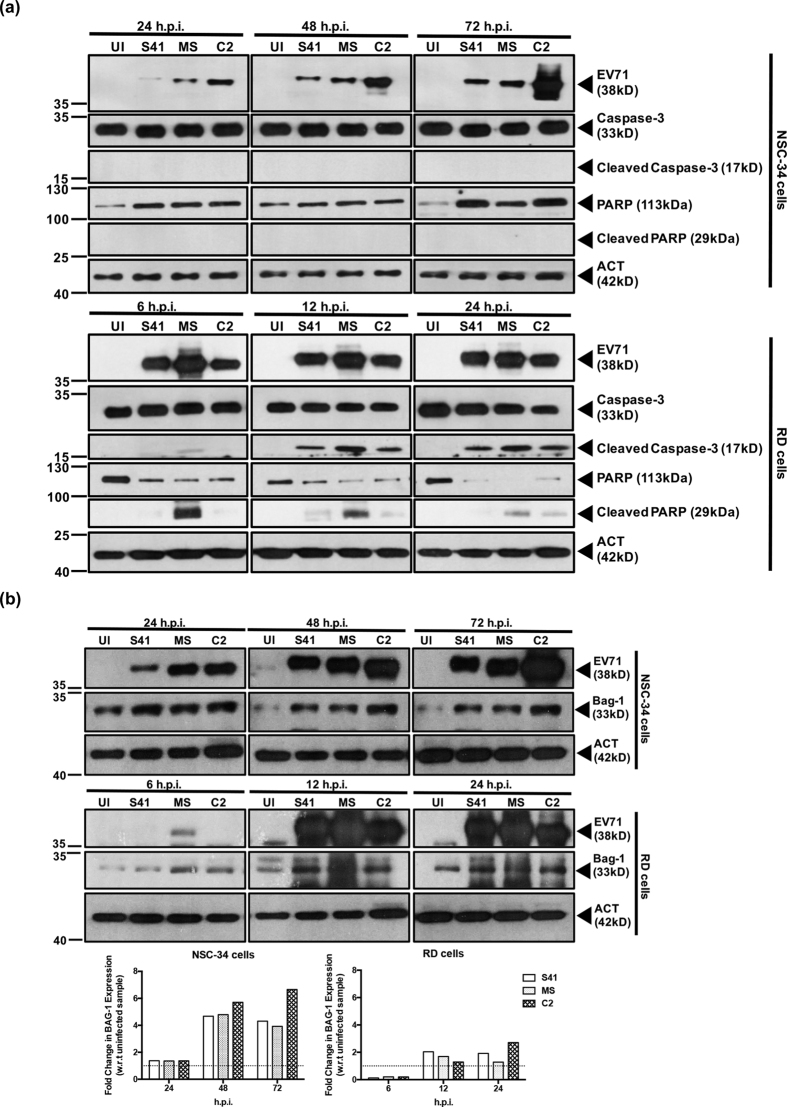
Caspase-3 and PARP cleavage, and expression of anti-apoptotic protein Bag1 in EV71-infected RD and NSC-34 cells. RD and NSC-34 cells were infected with S41, C2 and MS strains at MOI 1 and 10, respectively. At the indicated time points post-infection, Western blot analysis was performed on the cell lysates using antibodies specific to (**a**) full length or cleaved caspase-3 and PARP proteins or (**b**) anti-apoptotic protein Bag1. The band intensities were analyzed using ImageJ software, by normalizing against β-actin, and using the uninfected control (UI) as a reference (dotted line, fold change of 1). Gels images were cropped. These experiments were performed twice independently. One representative set is shown.

Furthermore, the infected cell lysates were analysed by Western blot for caspase and PARP cleavage, hallmarks of apoptotic events^40^. Signals for cleaved caspase-3 and PARP were detected in EV71-infected RD cells with increasing intensity over the course of infection (Fig. 5a). In contrast, no signal for both cleaved proteins was detected in EV71-infected NSC-34 cells (Fig. 5a), further confirming absence of caspase-dependent apoptosis.

Finally, absence of apoptosis in EV71-infected NSC-34 cells was further supported by the marked up-regulation of the anti-apoptotic marker Bag-1 relative to the uninfected control (Fig. 5b). In contrast, Bag-1 up-regulation in EV71-infected RD cells was more modest (Fig. 5b).

Absence of apoptosis seen with S41-infected NSC34 cells could be due to the lower virus susceptibility of these cells, as evidenced by the overall lower virus titers measured in the culture supernatants compared to RD cells (Fig. 1c). To address this possibility, S41 viral RNA transfection into NSC-34 cells was performed which led to comparable or even higher virus titer (109–1010 PFU/mL) (Fig. S6a) than the titers obtained in RD cells (Fig. 1c). However, no apparent CPE was observed at 48 and 72 hours post-transfection and the cells continued to divide (Fig. S6b). Furthermore, Western blot analysis of S41 RNA-transfected NSC-34 cells showed absence of caspase-3 and PARP cleavage, indicating that the cells did not undergo apoptosis (Fig. S6c). Finally, it was found that more than 80% of the NSC34 cells were infected (Fig. S7). Together, these data strongly argue against the hypothesis that absence of cell lysis and apoptosis in S41-infected NSC34 cells is due to lower infectivity and/or to a negligible percentage of infected cells.

Taken together these results indicate that unlike RD, SK-N-SH and SH-5YSY cells, NSC-34 cells do not undergo apoptosis upon EV71 infection, which suggests unique host-pathogen interactions in NSC-34 cells.

### Autophagy is induced in EV71-infected NSC-34 cells and is associated with release of virus particles in the culture supernatant

**Figure 6:**
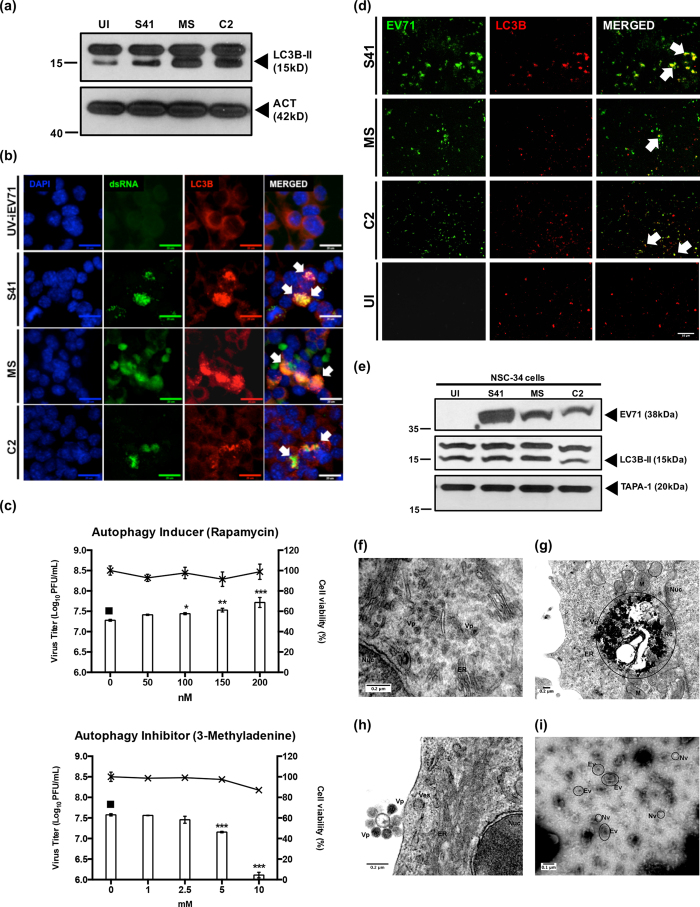
Autophagy in EV71-infected NSC-34 cells and detection of EV71-containing autophagic vacuoles in the culture supernatant. NSC-34 cells were infected with S41, C2 and MS strains at MOI 10 and at 48 h.p.i. (**a**) Western blot analysis was performed on the cell lysates using anti-LC3B primary antibodies. β-actin was used as the loading control. Gels images were cropped. (**b**) Immunostaining using antibodies specific to LC3B protein (red) and dsRNA (green). White arrows indicate co-localization of both signals. Images were taken at 60× magnification. Representative views are shown. Scale bar denotes 20 μm. UV-iEV71 infection served as negative control. (**c**) NSC-34 cells were treated with autophagy inducer (rapamycin) prior to S41-infection, or were treated post-infection with autophagy inhibitor (3MA). The culture supernatants were harvested at 48 h.p.i. for virus titer determination. Cytotoxicity of rapamycin and 3MA in NSC-34 cells was determined using alamarBlue™ cytotoxicity assay. Data are expressed as the mean ± SD of technical triplicates. Statistical analysis was performed using one-way ANOVA with Dunnett’s post-test (^*^p < 0.05, ^**^p < 0.005, ^***^p < 0.0005) against untreated cells (black square). (**d**) Immunostaining of exosome preparations using anti-VP1/VP0 (green) and anti-LC3B (red) antibodies. Arrows indicate co-localization of both signals. Uninfected cells (UI) served as negative control. Images were taken at 60× magnification. Scale bar denotes 10 μm. Representative views are shown. (**e**) Western blot analysis of the exosome preparations using anti-LC3B, anti-VP1/VP0 and anti-TAPA-1 primary antibodies. Gels images were cropped. (**f–h**) Transmission electron microscopy images of S41-infected NSC-34 cells (MOI 20) at 24 and 72 h.p.i. (positive staining). (**i**) Transmission electron microscopy images of exosome preparation from S41-infected NSC-34 culture supernatant (negative staining). Legend: Nuc, nucleus; M, mitochondria; V, vacuole; ER, endoplasmic reticulum; RC, replication complex; Vp, viral particles; Ev, enveloped virus; Nv, naked virus. Representative images are shown. These experiments were performed twice independently. One representative set is shown.

Apoptosis and autophagy have often been found to be inversely related, where induction of apoptosis is associated with inhibition of autophagy and vice versa^41^. The lack of apoptosis in EV71-infected NSC-34 cells therefore prompted us to determine whether autophagy may be induced instead. Consistently, increased signal intensity of the autophagy marker LC3B-II was seen in EV71-infected NSC-34 cells compared to the uninfected control (Fig. 6a). Furthermore, immunostaining revealed that LC3B protein co-localized with EV71 dsRNA which was not seen in NSC-34 cells infected with UV-inactivated EV71 (Fig. 6b). Finally, NSC-34 cells were treated with the autophagy inducer rapamycin prior to infection with S41 (pre-treatment), or were treated post-infection (post-treatment) with the autophagy inhibitor 3-methyladenine (3MA). Results showed increasing virus titers in the culture supernatant in the presence of increasing doses of rapamycin, and a dose-dependent decrease of the virus titers when using the autophagy inhibitor 3MA (Fig. 6c). These observations therefore support that induction of autophagy during EV71 infection of motor neuron derived NSC-34 cells enhances the production of infectious viral particles and that autophagosomes may be used as a scaffold for viral replication, as recently described in human muscle RD cells^42^.

Studies on poliovirus and coxsackie virus have reported non-lytic viral spread by making use of the host autophagic pathway, an exit mechanism termed autophagosome-mediated exit without lysis (AWOL)^43^,^44^. To investigate if autophagic vesicles are used by EV71 to exit NSC-34 cells, exogenous vesicles or exosomes present in the culture supernatant of EV71-infected NSC-34 were isolated. Immunostaining showed co-localization of the specific signals for LC3B and viral capsid VP1/VP0 in exosome preparations from EV71-infected NSC-34 culture supernatant (Fig. 6d). This was confirmed by Western blot analysis of these exosome preparations where the viral capsid protein VP0/VP1, autophagic marker LC3B, as well as general exosomal marker TAPA1^45^ were readily detected (Fig. 6e). Furthermore, exosome preparations were applied onto RD cell monolayers and a plaque assay was performed. Virus titers of 9.8, 6.6 and 7.4 log_10_ PFU/mL were obtained with S41, C2 and MS-derived exosome preparations, respectively, thus indicating that these exosome preparations contain infectious virus particles.

Finally, S41-infected NSC-34 cells were visualized by transmission electron microscopy (TEM). Positive staining allowed visualization of virus particles (Vp) in proximity of the endothelium reticulum (ER) inside the cells (Fig. 6f), and electron dense structures that resemble replication complexes could also be seen (Fig. 6g). Images of virus particles present at the cell surface were also captured (Fig. 6h). Furthermore, exosomes were isolated from the culture supernatant and processed for TEM using a negative staining approach, whereby virus particles and membranous structures appear less dense than the surrounding environment leading to inverted images traditionally obtained by positive staining method. The images revealed the presence of two types of vesicles that corresponds to naked (Nv) and enveloped (Ev) virus particles (Fig. 6i).

Together, results indicate that autophagy is induced in EV71-infected NSC-34 cells that helps both viral replication and non-lytic exit.

### S41, C2 and MS strains display differential ability to invade the CNS in AG129 mice

**Figure 7:**
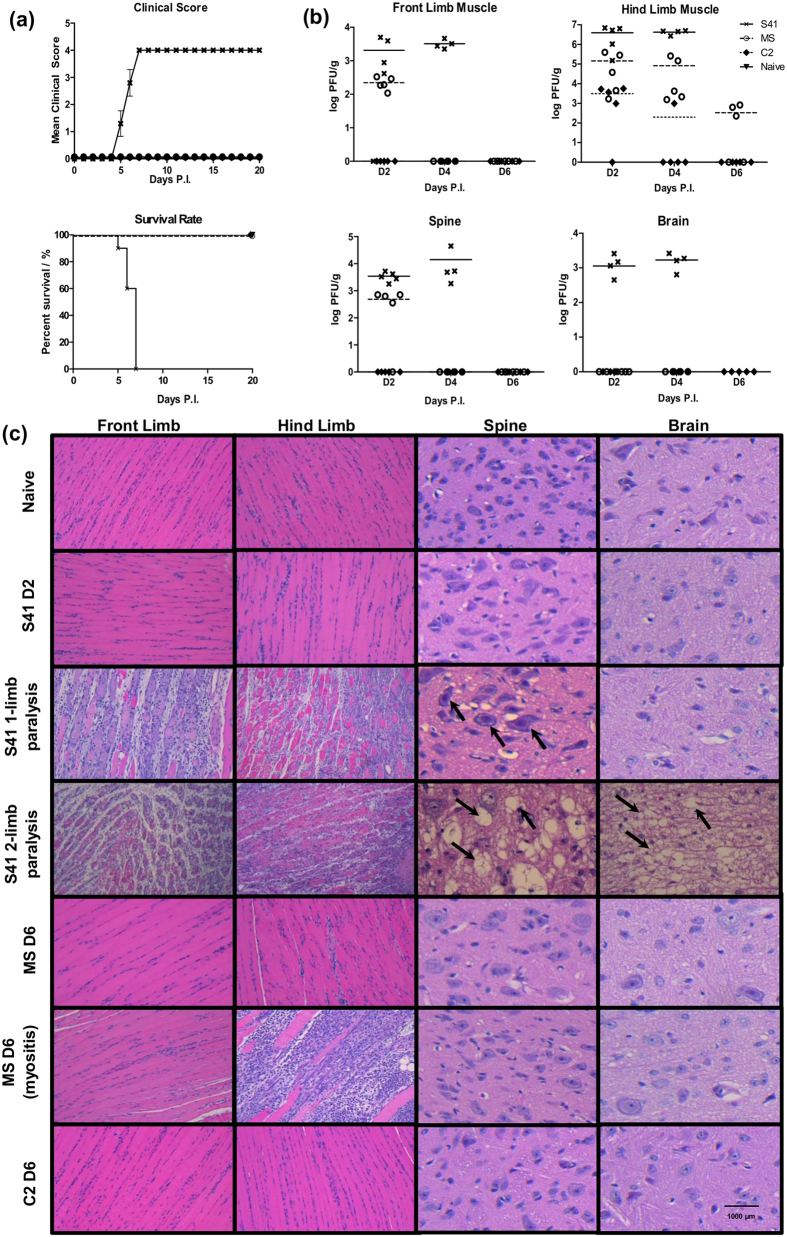
Survival rates, clinical scores, virus titers and histopathology in EV71-infected AG129 mice. Two-week old AG129 mice were infected with S41, MS or C2 strain at 10^7^ PFU/mouse via the intraperitoneal (i.p.) route. Naive mice received PBS instead. (**a**) Clinical scores and survival rate (n = 8–10 mice). The mice were monitored over a period of 20 days. Clinical scores were defined as follows: 0, healthy; 1, ruffled hair and hunchback appearance; 2, limb weakness; 3, paralysis of one limb; 4, paralysis of two limbs at which point the animals were euthanized. Data were expressed as mean ± SEM. A *p* value < 0.0001 was obtained for the log-rank test between S41-infected and naïve mice or C2- or MS-infected mice. Wilcoxon’s test showed that survival curves between S41-infected and naïve or C2- or MS-infected mice were significantly different (*p* value < 0.0005). (**b**) Virus titers in front limbs, hind limbs, spinal cord and brain from infected mice (n = 4–5) were determined at day 2, 4 and 6 p.i. by plaque assay. Individual values are shown. Means are represented by solid or dotted lines (refer to legend). (**c**) Histology analysis. Mice (n = 3) were euthanized at the indicated time points post-infection (MS and C2-infected mice) or upon observation of one or two limbs paralysis (for S41-infected mice). Paraffin sections of front limb, hind limb, spine and brain were stained with H&E and observed under light microscope. Black arrows indicate neuropil vacuolation and neuronal degeneration in the anterior horn region of the spinal cord and brainstem reticular formation of the brain from S41-infected mice. Myositis was observed in one MS-infected mouse at day 6 p.i. All observations were made at 20× magnification. Scale bar denotes 1,000 μm. Representative views are shown. All these experiments were performed two or three times independently. One representative set is shown.

Infection profiles of S41, C2 and MS strains were determined in 2-week old AG129 mice, a mouse model of EV71 infection previously established in our laboratory^20^. As previously reported^20^, S41-infected mice displayed progressive limb paralysis and succumbed to disease by day 5–7 p.i (Fig. 7a). In contrast, mice infected with C2 and MS strains remained asymptomatic and survived throughout the entire monitoring period (Fig. 7a). This observation suggests that neurovirulence is EV71 strain-dependent in the AG129 mouse model.

High virus titers were detected in the front and hind limb muscles at day 2 and 4 p.i., as well as in the spinal cord and brain from S41-infected mice (Fig. 7b). Note that in this experiment, S41-infected mice succumbed to infection by day 5 p.i and therefore no virus titer could be established at day 6 p.i. In contrast, C2 virus particles were only detected in the hind limb muscles of infected mice mostly at day 2 p.i. and for one out of five mice at day 4 p.i (Fig. 7b). MS virus was detected in the front and hind limb muscles at day 2, 4 and 6 p.i (Fig. 7b). Furthermore, significant amounts of MS virus were detected in the spinal cord at day 2 p.i but not at later time points (Fig. 7b). No MS and C2 virus was found in the brain of the infected animals at any of the time points assessed (Fig. 7b).

Histological analysis revealed that S41-infected mice displayed increasing limb muscle and CNS damage over time post-infection (Fig. 7c). At moribund stage when mice displayed two-limb paralysis, severe myositis in the limbs and severe CNS damage were observed as evidenced by neuropil vacuolation in the spinal cord and brainstem reticular formation of the brain (Fig. 7c). At the earlier stage of 1-limb paralysis, myositis was observed together with some damage in the spinal cord but not in the brain (Fig. 7c). In contrast, neither myositis nor CNS damage was observed with C2- and MS-infected mice over the course of infection, with the exception of one MS-infected mouse for which myositis was seen at day 6 p.i. but no CNS damage, which correlated with absence of limb paralysis (Fig. 7a). Interestingly, although a significant amount of MS virus particles were found in the spinal cord at day 2 p.i. (Fig. 7b), no tissue damage was observed (Fig. 7c).

Previous studies have identified a number of mutations in the viral genome that were shown to be involved in increased virulence in animal models, to confer a replicative advantage *in vitro*, or to correlate with CNS involvement in patients^22^,^46^,^47^,^48^,^49^,^50^,^51^,^52^,^53^,^54^,^55^,^56^,^57^. However, upon analysis of the viral genome of S41, C2 and MS, none of these mutations could explain the increased neurovirulence of S41 compared to C2 and MS in the AG129 mouse model. When comparing the nucleotide and amino acid sequences between neurotropic S41 strain and the myotropic MS/C2 strains, differences were found in the 5′and 3′UTR, VP2, VP3, 2 A, 3 A, 3 C, and 3D proteins (Table S1). The difference in VP2 at position 149 between S41 (I) and C2/MS (K) attracted our attention as the VP2-K149I mutation was previously reported to enhance the ability of EV71 to replicate in CHO (Chinese Hamster Ovary) cells, but did not lead to increased virulence in mice^49^. However, given that the mouse model used in the previous study is different from the AG129 mouse model, the possibility that this amino acid plays a role in the increased neurovirulence of S41 compared to MS and C2 strains, cannot be completely ruled out. The role and importance of the nucleotide and amino acid differences identified between S41 and C2/MS are currently under investigation.

Altogether, these data support that in the AG129 mouse model of EV71 infection, neurovirulence is strain-dependent, and clinical manifestations (limb paralysis) correlate with damage in both the spinal cord and brain. Furthermore, the data clearly indicate that S41 strain is more neurovirulent than C2 and MS strains, correlating with the superior ability of S41 to infect productively NSC-34 cells.

## Discussion

Since motor neurons terminate on skeletal muscles at the neuromuscular junctions, they represent the point of entry for EV71 to invade the CNS and reach the brainstem. We therefore studied and characterized the infection cycle of EV71 in the NSC-34 cell line, a motor neuron-like cell type^26^. Our data demonstrated that NSC-34 cells are permissive to EV71 infection, allowing the production of infectious viral particles in the culture supernatant. Infectivity was EV71 strain-dependent and correlated with the ability to invade the CNS *in vivo* and lead to neurological manifestations. Comparison of the amino acid sequence between neurovirulent S41 and myotropic C2and MS strains revealed a number of differences and their role in the phenotypic differences observed between these strains is under investigation.

While infection of NSC-34 cells with S41, MS and C2 strains led to detectable levels of infectious virus particles in the culture supernatants, significantly higher virus titers were measured with S41 compared to C2 and MS. Real-time PCR analysis indicated that the three virus strains were able to replicate within NSC-34 cells although with a lesser efficacy for MS strain. At the protein level, greater signal intensity for the VP1/VP0 capsid protein was clearly observed with C2- and MS-infected or transfected cell lysates compared to S41-infected or transfected cell lysates. These results suggested a possible impairment in egress ability for MS and C2 strains. A mutation in the γ_1_34.5 viral protein of herpes simplex virus 1 was found to interfere with viral egress, causing majority of the viral particles to accumulate inside the cytoplasm^58^. Similarly, mutations in the hepatitis C viral protein p7 were found to impair proton channel function which resulted in decreased viral egress^59^. While we have identified a number of differences in the viral genome of MS and S41 strains (data not shown), their contribution in viral egress efficacy needs to be further investigated.

Furthermore, upon transfection of NSC-34 cells with the corresponding purified viral genomes, similar C2 and S41 virus titer kinetics were obtained in the culture supernatant with the exception of the last time point (72 h.p.i.). This suggested that the superior virus production observed with S41 compared to C2 upon infection with the whole virus may be attributed to a differential ability of both virus strains to enter NSC-34 cells. We showed that virus entry into NSC-34 cells is not mediated by mSCRAB2 or mPSGL-1. The identity of EV71 receptor in NSC34 cells remains to be uncovered.

One of the most interesting and unexpected observations made with the NSC-34 infection model was the absence of cytopathic effect (CPE). This was in contrast to RD, SK-N-SH and SH-5YSY cells which displayed rapid and progressive CPE over time at MOI as low as 0.01. Absence of CPE in EV71-infected NSC-34 cells correlated with absence of apoptosis, again in sharp contrast with RD, SK-N-SH and SH-5YSY cells. We have rule out the possibility that absence of apparent lysis and apoptosis in EV71-infected cells could be due to low infectivity or small percentage of infected cells. EV71 infection has previously been reported to trigger apoptosis in many different cell types including lymphocytes^60^, endothelial cells^61^, muscle cells and neural cells^38^,^62^. The apoptosis pathway that is induced appears to be cell-type specific whereby EV71-induced apoptosis can be either caspase-dependent intrinsic apoptosis or calpain-induced caspase-independent apoptosis^63^. Proteases 2 A and 3 C were demonstrated to cleave PARP in RD cells^64^. We found that neither caspase-3 nor PARP were cleaved in EV71-infected NSC-34 cells. This phenomenon likely results from specific interactions (or the lack thereof) between viral proteins and host factors. Further work is needed to address this aspect.

Instead, anti-apoptotic and autophagic pathways were markedly induced in EV71-infected NSC-34 cells. Our data support that autophagy vesicles are being used both as scaffold for virus replication and non-lytic exit of the virus particles. While the former mechanism (autophagy vesicles used as replication scaffold) has already been reported in RD cells^42^,^64^, the latter (using autophagy vesicles for non-lytic exit) has not been described for EV71 so far. However, recent studies have shown that other members of the *Picornaviridae* family including Hepatitis A virus, coxsakievirus B and poliovirus are able to spread in a non-lytic fashion, challenging the traditional view according to which viruses are classified in a mutually exclusive way as either lytic or non-lytic. Hepatitis A virus has been reported to be able to switch between two forms, namely a naked form which is more stable and is shed in feces which facilitates transmission; and a quasi-enveloped form, as a result of its interaction with host proteins associated with endosomal-sorting complexes required for transport (ESCRT) during biogenesis, cloaking the virus in host membranes and leading to quasi-enveloped virus particles that escape antibody-mediated neutralisation due to the lack of viral proteins at their surface^65^. Coxsackie B3 has been reported to be released in membranous microvesicles with the autophagosomal marker LC-3 protein^66^. Poliovirus has been shown to exit its host cell via autophagosome-mediated exit without lysis (AWOL), leading to the release of single-membraned phosphatidylserine (PS) rich vesicles^67^,^68^. These vesicles were shown to improve viral infection as compared to free poliovirus virions, possibly due to the fact that together with CD155, the poliovirus receptor, PS was found to act as a cofactor required for viral entry^68^. Therefore, given that the autophagy pathway has been proposed to prevent cellular damage in neurons^69^, one could speculate that absence of apoptosis in EV71-infected motor neurons is meant to protect the integrity of these important cells, and at the same time facilitate virus transmission to the brain. This phenomenon has been previously described for rabies virus^70^, as well as for Theiler’s virus, another member of the *Picornaviridae* family^71^.

In conclusion, we have shown that the motor-neuron NSC-34 cells can be infected productively with EV71 strains. We report here for the first time a non-lytic exit pathway for EV71 using autophagic vesicles, which may facilitate viral dissemination throughout the CNS, as previously reported for other members of the *Picornaviridae* family. Furthermore, the infectivity in NSC-34 cells seems to correlate with the ability to invade the CNS of AG129 mice. Thus, the combined usage of the AG129 mouse model and NSC-34 motor neuron cell line may help in the study of intrinsic neurovirulence potential of EV71 strains.

## Materials and Methods

Detailed protocols are available in the Supplemental Experimental Procedures.

### Ethics statement

The animal experiments described in this work were carried out according to the guidelines of the National Advisory Committee for Laboratory Animal Research (NACLAR) in the AAALAC-accredited NUS animal facilities (http://nus.edu.sg/iacuc/). They were approved by the NUS Institutional Animal Care and Use Committee (IACUC) under protocol numbers 070/10 and 139/12. Non-terminal procedures were performed under anaesthesia, and all efforts were made to minimize suffering.

### Cell Culture and Virus Strains

All the cell lines were maintained according to ATCC recommendations. The EV71 strains used in this study (Table S2) were propagated in RD cells (S41, C2 and MS strains) or in NIH/3T3 (EV71:TLLm). UV-inactivated EV71 (UV-iEV71) was obtained by subjecting the virus to ultraviolet light irradiation for 2 hours.

### Viral Infection of Cell Lines for Virus Kinetic Studies

Cells were infected with the EV71 strains at various multiplicity of infection (MOI) for 1 hour. The cell monolayers were then washed twice before incubation in complete culture medium. At various time points post-infection, the cells or supernatants were harvested and processed for further analyses.

### Plaque Assay

RD or NIH3/T3 cells were infected with serially diluted viral culture supernatants for 1 hour incubation. The cell monolayer was then washed before addition of overlay medium. After 3–4 days incubation, the wells were washed and crystal violet was added. Virus titers were expressed as PFU/mL.

### Quantitative Real Time PCR

Cells were infected with EV71 at MOI 1 or 10. At various time points post-infection, the cells were washed, trypsinized and gently pelleted by centrifugation. Total RNA was extracted using the TRizol method. RNA was further purified using the RNeasy mini kit and treated with DNAse I. Complementary DNA (cDNA) was obtained and real-time PCR was performed using the iTaq Universal SYBR green supermix and EV71 VP1 specific primers (Table S3). Intracellular viral RNA titers were normalized to GAPDH RNA levels and expressed as fold change (EV71 expression/GAPDH expression).

### Viral RNA Transfection

Viral RNA genome was extracted using the QIAamp Viral RNA Mini Kit and mixed with Lipofectamine 2000 prior to adding to the NSC-34 cell suspension. At various time points of incubation, the culture supernatant was harvested for virus titer determination by plaque assay, while the cell pellet was harvested for Western blot analysis.

### Cellular Cytotoxicity Assay

Cells were incubated with alamarBlue reagent for 3 hours. Fluorescence was read at the excitation wavelength of 570 nm and emission wavelength of 585 nm. The percentage of cell viability was compared against uninfected cells.

### Western Blot Analysis

Total proteins were extracted from whole cell lysates and quantified by Bradford Protein Assay. Heat-denatured proteins were separated onto SDS-PAGE prior to transferring onto nitrocellulose membrane. The primary and secondary antibodies employed are listed in Table S4. The bands were visualized on X-ray films. Densitometric quantification was performed using ImageJ v1.48 freeware (http://rsbweb.nih.gov/ij/index.html) and the relative band intensity for each protein of interest was normalized against β-actin.

### mSCARB2 Antibody Blocking Assay

Cells were pre-incubated with anti-mouse SCARB2 antibody or with IgG control for 1 hour. The cells were then washed twice before infection with EV71 strains. At 48 or 24 h.p.i., the culture supernatants were harvested for virus titer determination by plaque assay.

### mSCARB siRNA-mediated Gene Silencing

NSC-34 cells (10^5^) were reverse transfected with various concentrations of mSCARB-2 targeting siRNAs for 48 hours. The cells were then washed twice before infection with EV71 S41 strain at MOI 10. At 48 h.p.i., the culture supernatant was harvested for virus titer determination by plaque assay.

### Indirect Immunofluorescence Assay (IFA)

NSC-34 or RD cells were seeded on coverslips, infected with EV71 strains for 1 hour and fixed with ice-cold methanol at various time points post-infection. The fixed cells were incubated with primary (anti-dsRNA, anti-EV71, or anti-LC3B) antibodies followed by incubation with secondary antibodies. Cell nuclei were stained with DAPI. The exosomes samples were air dried on coverslip prior to fixation and immunostaining. The samples were viewed using conventional optical-fluorescence microscope (Olympus IX81).

### Annexin-V Apoptosis Assay

At various time points post-infection, the cells were harvested for Annexin-V and propidium iodide (PI) staining using the Annexin V FITC Apoptosis Detection kit I (BD Pharmingen). Mock-infected cells and NSC-34 cells pre-treated with staurosporine served as negative and positive controls, respectively. The cell suspensions were analysed by FACS.

### TUNEL Assay

At various time points post-infection, the infected cells were fixed with methanol before TUNEL assay was carried out using the TiterTacs Colorimetric Apoptosis Detection Kit (Trevigen) following the manufacturer’s instructions.

### Rapamycin and 3-Methyladenine (3MA) Treatments

For autophagy induction pre-treatment assay, NSC-34 cells were pre-incubated with rapamycin for 3 hours before infection with S41 virus. For autophagy inhibition post-treatment assay, NSC-34 cells were infected with S41 prior to addition of 3MA into the culture supernatant. In both assays, the culture supernatants were harvested at 48 h.p.i. for virus titer quantification by plaque assay.

### Exosomes Isolation

Clarified culture supernatants from EV71-infected NSC-34 cells were harvested at 48 h.p.i. and were incubated with exosome isolation reagent (Life Technologies) prior to centrifugation. The pellet, which is enriched in exosomes, was resuspended in sterile PBS for further analysis.

### Transmission Electron Microscopy

S41-infected culture supernatant and cells were fixed with glutaraldehyde and osmium tetraoxide. The fixed cells were then dehydrated in ascending graded series of ethanol and embedded with Low Viscosity Epoxy Resin. The exosome preparations were fixed with osmium tetroxide and negatively stained with phosphor-tungstic acid and uranyl acetate. All samples were viewed under transmission electron microscope, JEM-1010 (JEOL, Japan).

### Mouse Model of EV71-infection

EV71 infection of two-week-old AG129 mice was carried out via the intraperitoneal (i.p.) route. Mice were observed daily for a period of twenty days. Clinical disease and symptoms were graded as follows: 0, healthy; 1, ruffled hair and hunchbacked appearance; 2, limb weakness; 3, paralysis in one limb; 4, paralysis in two limbs; and 5, death. Upon observation of two-limb paralysis, animals were promptly euthanized for ethical reasons.

### Histology of EV71-infected Mice

Mice were euthanized at the indicated time points post-infection, or upon observation of one- or two-limb paralysis. Following systemic perfusion with PBS and neutral buffered formalin (NBF), the hind limbs, front limbs, spinal cord and brain were harvested, processed, paraffin embedded, sectioned and stained with hematoxylin and eosin (H&E). Analysis of the sections was performed on blinded samples.

### Determination of Virus Titers in Organs from Infected Mice

EV71-infected AG129 mice were euthanized and perfused systemically prior to harvesting the front and hind limb muscles, spinal cord and brain. The organs were weighed before homogenization. Plaque assay was performed on the clarified homogenates. Virus titers were expressed as PFU per gram of tissue.

### Statistical Analysis

All statistical analyses were carried out using Graphpad Prism software (GraphPad Software, Inc). Data are expressed as mean ± standard deviation (SD), or mean ± standard error of the mean (SEM). Kaplan-Meier survival curves and clinical score curves were analysed using a log rank test and the Wilcoxon test, respectively.

## Additional Information

**How to cite this article**: Too, I. H. K. *et al*. Enterovirus 71 infection of motor neuron-like NSC-34 cells undergoes a non-lytic exit pathway. *Sci. Rep.* **6**, 36983; doi: 10.1038/srep36983 (2016).

**Publisher’s note:** Springer Nature remains neutral with regard to jurisdictional claims in published maps and institutional affiliations.

## Supplementary Material

Supplementary Information
