# Supplementary material for: Enterovirus 71 infection of motor neuron-like NSC-34 cells undergoes a non-lytic exit pathway
Source: Sci Rep. 2016 Nov 16;6:36983. doi: 10.1038/srep36983 (PMC5111112; doi:10.1038/srep36983)
Supplement: Supplementary Information [file srep36983-s1.doc]

**Supplemental information**

**Title: Enterovirus 71 infection of motor neuron-like NSC-34 cells undergoes a non-lytic exit pathway**

Authors: Issac Horng Khit TOO^1,2#^, Huimin YEO^1,2#^, Benedict YAN^3^, Eshele Anak LIBAU^1,2^, Josephine L.C. HOWE^1^, Ze Qin LIM^1,2^, Shalini D/O SUKU-MARAN^4,5^, Wei-Yi ONG^4,5^, Kaw Bing CHUA^6^, Boon Seng WONG^5,7^, October Michael SESSIONS^8^, Vincent T. K. CHOW^1^, Sylvie ALONSO^1,2*^

Affiliations: ^1^Department of Microbiology & Immunology, Yong Loo Lin School of Medicine and ^2^Immunology Programme, Life Sciences Institute, CeLS building, 28 Medical Drive, National University of Singapore, Singapore 117456. ^3^Department of Laboratory Medicine, 5 Lower Kent Ridge Road, National University Hospital, Singapore 119074. ^4^Department of Anatomy, Yong Loo Lin School of Medicine and ^5^Neurobiology and Ageing Programme, Life Sciences Institute, CeLS building, 28 Medical Drive, National University of Singapore, Singapore 117456. ^6^Temasek Life Sciences Laboratory, 5A Engineering Drive 1, National University of Singapore, Singapore 117411. ^7^Department of Physiology, Yong Loo Lin School of Medicine, CeLS building, 28 Medical Drive, National University of Singapore, Singapore 117456. ^8^Program in Emerging Infectious Diseases, Duke-NUS Graduate Medical School, 8 College Road, Singapore 169857.

^#^ Both authors contributed equally to the work.

*Corresponding author (SA): Email: [micas@nus.edu.sg](mailto:micas@nus.edu.sg)

| **(a)** | **(b)** |
| --- | --- |
| **(c)**  **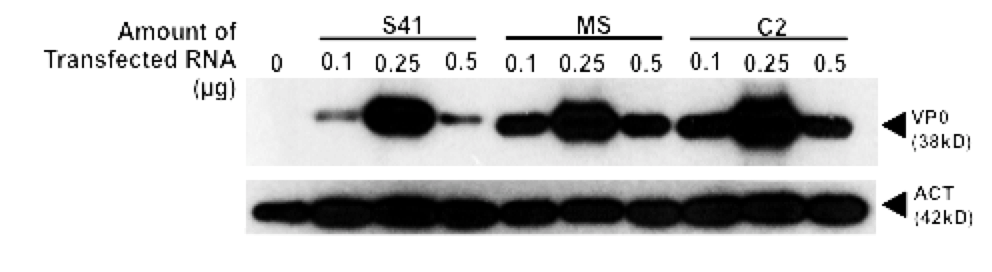** | **(d)** |

**Figure S1. Transfection of EV71 RNA into NSC-34 cells.**

NSC-34 cells were transfected with various amounts (0.1, 0.25, and 0.5 µg ) of purified S41, MS and C2 viral RNA, or with transfection controls. (a) At 48 hours post-transfection, cell viability was determined using alamarBlue cytotoxicity assay. The 70% cell viability threshold is indicated by a dotted horizontal line. (b) The virus titer in the culture supernatants was determined by plaque assay. Data are expressed as the mean ± SD of technical triplicates. (c-d) Western blot anlaysis of the cell lysates using anti-VP0/VP1 primary antibodies. The bands intensities were analysed using ImageJ software, by normalizing against β-actin. Gels images were cropped. N.D., no detection. These experiments were performed twice independently. One representative set is shown.

| **(a)** | **(b)** |
| --- | --- |
| 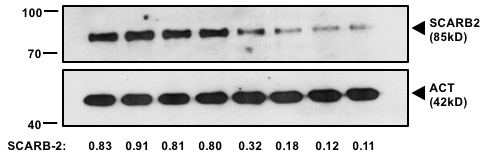**(c)** | 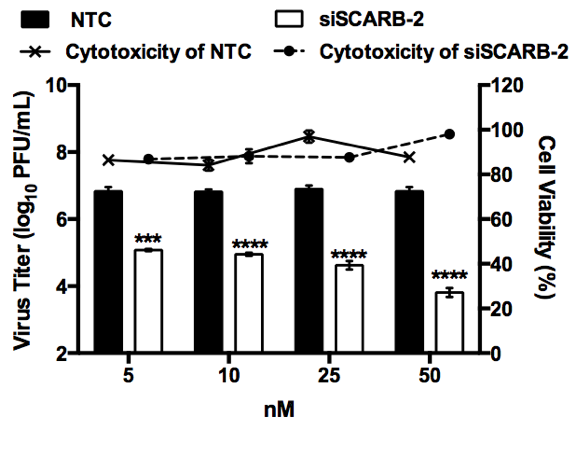**(d)** |

**Figure S2. mSCARB-2-blocking and silencing assays in NSC-34 cells.**

(a&b) NSC-34 cells were pre-treated with various concentrations of mSCARB-2 specific antibodies as indicated, or with IgG isotype control, prior to infection with (a) mouse-adapted EV71:TLLm strain at MOI 1, or with (b) S41, MS and C2 strains at MOI 10. Virus titers were determined by plaque assay at 72 h.p.i.. Cell viability was monitored using the alamarBlue™ cytotoxicity assay. Statistical analysis was performed using one-way ANOVA with Dunnett’s post-test (* *p*<0.05, ** *p*<0.005, *** *p*<0.0005) against untreated cells (black square). (c&d) siRNA-mediated SCARB-2 silencing of NSC-34 cells. NSC-34 cells (10^5^ cells) were transfected with SCARB-2 siRNA at various concentrations for 48 h.p.t. (c) The efficiency of siRNA knockdown was verified by Western blot. Relative band quantification (below Western blot) was determined by ImageJ, by normalizing to loading control, β-actin. After siRNA transfection, SCARB-2-knockdown cells were infected with EV71 S41 at MOI 10. (d) Virus titers were determined by plaque assay at 48 h.p.i. Non-targeting siRNA (NTC) served as control. The cell viability of transfected cells was assessed using alamarBlue™ viability assay. Statistical analysis two-tailed student’s t-test (* *p*<0.05, ** *p*<0.005, *** *p*<0.005, **** *p*<0.0001). Data are expressed as the mean ± SD of technical triplicates. These experiments were performed twice independently. One representative set is shown.

|  |
| --- |
| **Figure S3. Immunostaining of S41-infected RD and NSC-34 cells.**  (a) RD (10^5^ cells) and NSC-34 cells (1.5x10^5^ cells) were seeded on coverslip and infected with S41 at MOI 1 and 10, respectively. At the indicated time points post-infection, the cells were fixed with methanol and stained with anti-EV71 antibodies (VP1) (viral signal, red). Nuclei were DAPI stained. (b) Corrected total cell fluorescence (CTCF) of each cell was quantified using ImageJ based on three random microscopic views. Cells with higher viral fluorescence intensity than uninfected cells were counted as positive (infected) cells. The percentage of infected cells was computed with the equation: (Infected cells/ Total cells) x 100%. Error bars represent mean 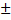 standard deviation of the three microscopic views. Scale bar denotes 50µm. Images were taken using Olympus IX81. |

**
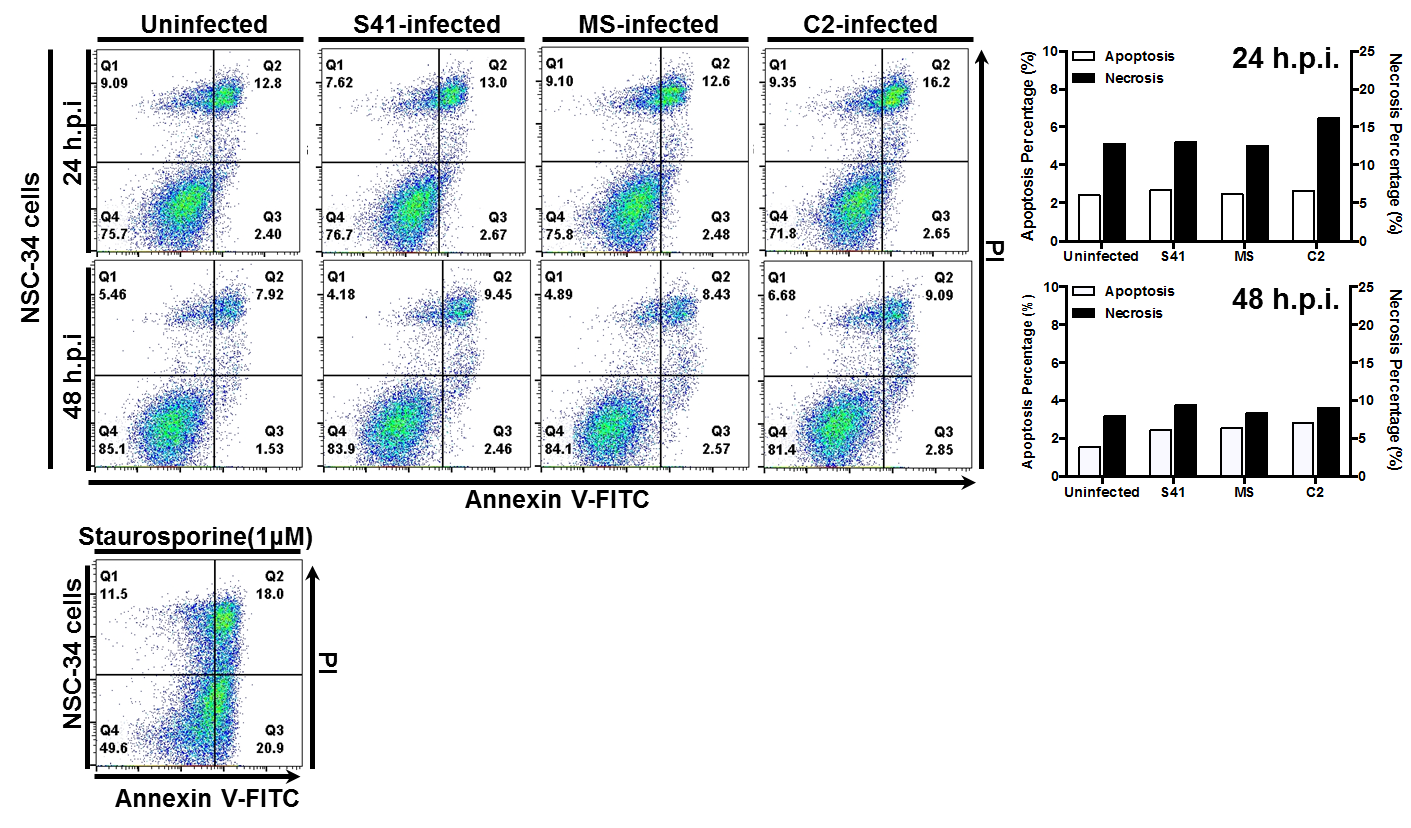

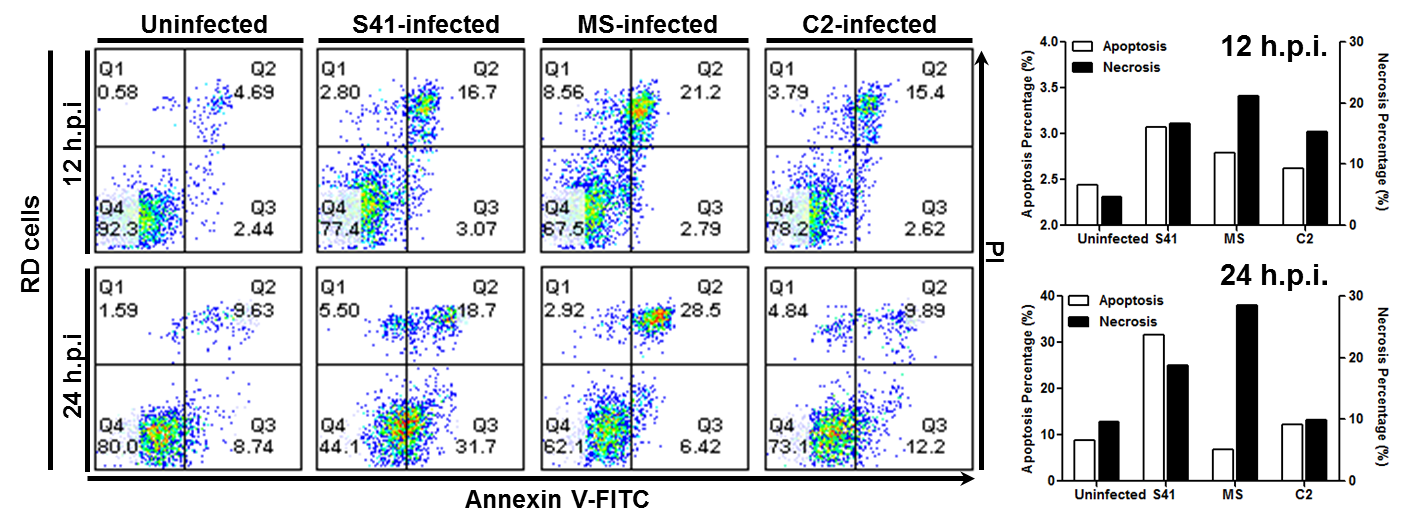
**

**Figure S4. Scatter plots for Annexin/Propidium iodide staining (fig 4A)**

RD and NSC-34 cells were infected with S41, C2 and MS strains at MOI 1 and 10, respectively. At the indicated time points post-infection, the cells were harvested and stained for Annexin V and Propidium Iodide, prior to FACS analysis. Staurosporine-treated NSC-34 cells were used as positive control. Legend: Q1, necrotic cells; Q2, damaged cells; Q3, apoptotic cells; Q4, viable cells.

**Figure S5. TUNEL assay of EV71-infected cells.**

SK-N-SH, SH-SY5Y and NSC-34 cells were infected with S41, MS and C2 at the indicated MOI. At 36 h.p.i. (SK-N-SH and SH-SY5Y cells) or 72 h.p.i. (NSC-34 cells), the cells were fixed and TUNEL assay was carried out. Positive apoptotic control cells were generated by incubation with TACs nuclease (Trevigen). Data are expressed as the mean ± SD of technical triplicates. Statistical analysis was performed using two-way ANOVA test with Bonferroni correction against mock-infected samples. (* *p*<0.1, ** *p*<0.01, *** *p*<0.001). This experiment was performed twice independently. One representative set is shown.

| **(a)** | 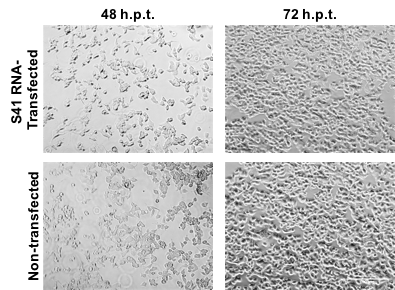**(b)** |
| --- | --- |
| **(c)**  **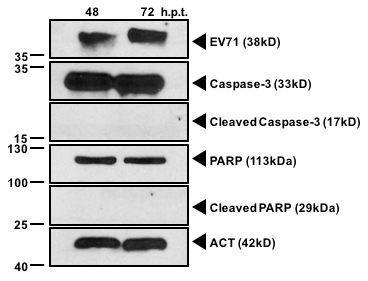** | |
| **Figure S6. S41 viral genome transfection into NSC-34 cells.**  2.5μg of EV71 S41 viral genome were transfected into NSC-34 cells (10^5^ cells). (A) Culture supernatant was harvested at 48 and 72 h.p.t. for viral titer determination by plaque assay. (B) Phase-contrast microscopic images of S41-transfected NSC-34 cells at various time points post-transfection. Non-transfected cells served as control. Scale bar represents 100μm. (C) Cell lysates were subjected to Western blot analysis using antibodies specific to VP1, and cleaved caspase-3 and PARP proteins. β-actin detection served as loading control. Representative of two biological repeats is shown. | |

**Figure S6. S41 viral genome transfection into NSC-34 cells.**

0.25 µg of S41 viral genome transfected into NSC-34 cells (10^5^ cells). At 48 and 72 h.p.t., the cells were fixed with methanol and probed for viral protein (anti-VP1 antibodies). Nuclei were DAPI stained. Corrected total cell fluorescence (CTCF) of each cell was quantified using ImageJ based on three random microscopic views. Cells with higher viral fluorescence intensity than uninfected cells were counted as positive (infected) cells. The percentage of infected cells was computed with the equation: (Infected cells/ Total cells) x 100%. Error bars represent mean
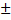
 standard deviation of the three microscopic views. Scale bar denotes 50µm. Images were taken using Olympus IX81.

| **Table S2. EV71 strains used in this study.** | | | | | |
| --- | --- | --- | --- | --- | --- |
| **Strain** | **Subgenogroup** | **Accession Number** | **Year of isolation** | **Clinical symptoms in patient** | **Ref.** |
| Strain 41 (S41) (5865/SIN/000009) | B4 | AF316321 | 2000 | Encephalitis, pulmonary edema, interstitial pneumonitis | 1 |
| MS Strain (MS) (EV71/7423/MS/87) | B2 | U22522 | 1987 | Acute flaccid paralysis, neurovirulent | 2 |
| C2 Strain (C2) (NUH0075/SIN/08) | C2 | FJ172159 | 2008 | Mild HFMD, papules | 3 |
| EV71:TLLm  (Serially passaged from EV71:BS, KF514878, in NIH/3T3 cells) | B4 | KF514879 | 2014 | Brain stem infection | 4 |

| **Table S3. Primers used in this study.**  Primers efficiencies were within 90-110%. | | |
| --- | --- | --- |
| **Target Gene** | **Forward Primer** | **Reverse Primer** |
| VP1 (S41) | GCACAGGTCTCAGTTCCGTT | CACGCCTGACATGCTTCAT |
| VP1 (C2) | ACAGGCCAAAACACGCAGGT | TTCGGGTTGGTTGTGCCTTC |
| VP1 (MS) | GCGCTCCAAGCTGCTGAAAT | GCTCGACTTTCCTGCGCATC |
| GAPDH | CATCATCTCCGCCCCTTCTG | TGGTCATGAGCCCTTCCACA |

| **Table S4. Primary and secondary commercial antibodies used in this study for Western blot analysis.** | | |
| --- | --- | --- |
| **Proteins of Interest** | **Primary Antibody** | **Secondary Antibody** |
| VP0/VP1-EV71 | Mouse anti-EV71 antibody (MAB979, 1:1,000 dilution, Merck) | Anti-mouse HRP-conjugated antibody (#172-1011, 1:5,000 dilution, Bio-Rad) |
| Mouse SCARB2 | Goat anti-mouse SCARB2 antibody (AF1888, 1:1,000 dilution, R&D systems) | Anti-goat HRP antibody (#172-1034, 1:5,000 dilution, Bio-Rad) |
| Full length caspase 3 | Rabbit anti-Caspase 3 antibody (ab32351, 1:5,000 dilution, Abcam) | Anti-rabbit HRP antibody (#170-6515, 1:5,000 dilution, Bio-Rad) |
| Cleaved caspase 3 | Rabbit anti-cleaved Caspase 3 antibody (ab3623, 1:100 dilution, Abcam) | Anti-rabbit HRP antibody (#170-6515, 1:5000 dilution, Bio-Rad) |
| Full length PARP | Rabbit anti-PARP antibody (ab 32138, 1:3000 dilution, Abcam) | Anti-rabbit HRP antibody (#170-6515, 1:5,000 dilution, Bio-Rad) |
| Cleaved PARP | Rabbit anti-cleaved Caspase 3 antibody (04-576, 1:10,000 dilution, Abcam) | Anti-rabbit HRP antibody (#170-6515, 1:5,000 dilution, Bio-Rad) |
| LC3B | Rabbit anti-LC3B antibody (ab51520, 1:3,000 dilution, Abcam) | Anti-rabbit HRP antibody (#170-6515, 1:5,000 dilution, Bio-Rad) |
| Beta actin | Rabbit anti-beta actin antibody (ab8227, 1:20,000 dilution, Abcam) | Anti-rabbit HRP antibody (#170-6515, 1:5,000 dilution, Bio-Rad) |

**Supplemental Experimental Procedures**

**Cell Culture and Virus Strains.**

NSC-34 mouse motor neuron-like hybrid cells (Cellutions Biosystems, CLU140), NIH/3T3 mouse fibroblasts cells (ATCC® CRL-1658™), human rhabdomyosarcoma (RD) cells (ATCC® CCL-136™), SK-N-SH (ATCC® HTB-11™) and SH-SY5Y (ATCC® CRL-2266™) human neuroblastoma cells were employed in this study. SK-N-SH and SH-SY5Y were kindly provided by Dr. J. J. H. Chu (Department of Microbiology, National University of Singapore, Singapore) and Prof. B.L. Tang (Department of Biochemistry, National University of Singapore, Singapore), respectively. All cell lines were maintained in Dulbecco's Modified Eagle's medium (DMEM) (Gibco) supplemented with 10% fetal bovine serum (FBS) (Gibco) at 37ºC in a 5% CO_2_ humidified incubator. The Enterovirus 71 strains used in this study were non-mouse-adapted EV71 S41 (5865/SIN/00009, Accession No.: AF316321), MS (EV71/7423/MS/87, Accession No.: U22522), C2 (NUH0075/SIN/08, Accession No.: FJ172159) and EV71:TLLm which was obtained after serial passages in NIH/3T3 cells of EV71:BS (Accession No.: KF514878) (Table S2). S41, MS and C2 were propagated in RD cells, while EV71:TLLm was passaged in NIH/3T3 cells. Virus titers were determined by plaque assay (section below) on RD (S41, MS and C2) or NIH/3T3 (EV71:TLLm) cells and concentrations were expressed as plaque-forming units (PFU) per mL (PFU/mL).

**Quantitative Real Time PCR.**

Cells were seeded onto T25-flasks at a density of 5
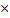
 10^6^ cells/flask and infected with EV71 at MOI 1 or 10. At 6, 12, 24, 48, 72 and 96 h.p.i, the supernatants were removed, and the cells were washed twice with PBS. Upon trypsinisation, cells were washed thrice with DMEM. Cells were gently pelleted by centrifugation. Viral RNA was extracted via the phenol-chloroform method. Briefly, 1 mL of TRIzol reagent (Invitrogen) was added to the cell pellet. Chloroform (200 μL) was added and the samples were mixed thoroughly before being incubated at room temperature (RT) for 3 minutes. Samples were then centrifuged at 12,000 r.p.m. for 15 minutes at 4°C to obtain the RNA-containing aqueous phase. Isopropanol was added to precipitate RNA from the aqueous phase. RNA was isolated using the RNeasy mini kit (Qiagen). Following manufacturer’s instructions, the RNA pellet was eluted from the column using nuclease-free water. RNA was treated with DNAse I (Sigma-Aldrich) to remove genomic DNA and then quantitated using Nanodrop. Next, complementary DNA (cDNA) was synthesized from RNA using iScript cDNA synthesis kit (Bio-rad). The PCR was then carried out using a 7500 Real-time PCR system (Applied Biosystems). The iTaq Universal SYBR green supermix (Bio-Rad) was used in the relative real-time SyBr green RT-PCR quantification assay of EV71 VP1 region, using GAPDH as an internal control (primers listed in Table S3). 40 cycles of amplification were completed with each cycle consisting of 95°C for 15 seconds and 60°C for 1 minute. Intracellular viral RNA titers were normalized to GAPDH RNA levels in each sample and expressed in terms of fold change (EV71 expression/GAPDH expression).

**Viral RNA Transfection.**

Viral RNA genome (10^8^ PFU/mL) from extracted from EV71 S41, MS and C2 using the QIAamp Viral RNA Mini Kit (Qiagen), according to the manufacturer’s instructions. The viral RNA was diluted to various concentrations (0.1, 0.25 and 0.5 µg) with OptiMEM (Invitrogen) in a total volume of 50 µL and incubated with OptiMEM for 5 minutes at RT. After 5 minutes incubation, 1 µL of Lipofectamine 2000 (Invitrogen) was mixed with 49 µL of OptiMEM and added to the RNA mixture, prior to 30 minutes incubation at RT. NSC-34 cell suspension (10^5^ cells in 400 µL) was mixed with 100 µL of transfection mixture and added into each well. The cells were then incubated for various time points. For each time point, the culture supernatant was collected for viral determination by plaque assay, while the cell pellet was harvested for Western blot analysis. The cytotoxicity of RNA and transfection reagents was measured using the alamarBlue (Invitrogen) cytotoxicity assay (section below).

**Cellular Cytotoxicity Assay.**

RD (5 × 10^4^ cells), SK-H-SH (5 × 10^4^ cells), SH-SY5Y (5 × 10^4^ cells), NSC-34 (5 × 10^4^ cells) and NIH/3T3 (2.5 × 10^4^ cells) seeded on 96-well plates were washed twice before adding 1× alamarBlue reagent (Invitrogen) diluted in 2% FBS-supplemented DMEM and incubated at 37°C in the presence of CO_2_ for 3 hours. Fluorescence was captured using the microplate reader (Infinite 200, Tecan) at the excitation wavelength of 570 nm and emission wavelength of 585 nm. The percentage of cell viability was compared against uninfected cells.

**Western Blot Analysis.**

The proteins from total cell lysates were extracted using Mammalian Protein Extraction Reagent (M-PER) (Pierce) supplemented with 1% Halt Protease Inhibitor Cocktail and 1% 0.5 M EDTA solution (Pierce). Protein quantification was performed using Quick Start™ Bradford Protein Assay (Bio-Rad). Heat-denatured proteins (5 μg) were loaded into 10% SDS-polyacrylamide gel and resolved at constant voltage of 100 V for 2.5 hours, before transferring onto nitrocellulose membrane (Bio-Rad) using Trans-Blot® Turbo™ Transfer System (Bio-Rad) at 17 V for 7 minutes. After blocking for 1 hour at RT with 5% w/v blocking reagent (Bio-Rad) in 1× Tris-buffered saline with Tween-20 (TBST) buffer, the membrane was probed with specific primary antibodies (Table S4) overnight at 4°C before subjecting to 1 hour incubation with horseradish peroxidase-conjugated anti-mouse or anti-rabbit IgG (Table S3) at RT. The membrane was then visualized using Clarity™ ECL Western Blotting substrate (Bio-Rad) on the X- ray film. The densitometric quantification was performed using ImageJ v1.48 freeware (<http://rsbweb.nih.gov/ij/index.html>) and the relative band intensity for each protein of interest was normalized against β-actin.

**mSCARB2 Antibody Blocking Assay.**

NSC-34 (2×10^5^ cells) and NIH/3T3 (10^5^ cells) were seeded on 24-well plates overnight, prior to pre-incubation with anti-mouse SCARB2 antibody (R&D systems, AF1888) at the concentration of 0, 5, 15, 30 µg/mL, or with IgG control (R&D systems, AF108C) at 30 µg/mL for 1 hour at 37ºC. The cells were washed twice before infection with S41, MS and C2 (NSC-34 cells), and EV71:TLLm (NIH/3T3 cells) at MOI 10 and 1 for 1 hour, respectively. At 48 (NSC-34 cells) and 24 (NIH/3T3 cells) h.p.i., the culture supernatant was collected for virus titer determination by plaque assay. Cytotoxicity of the SCARB-2 antibodies and IgG control in NSC-34 cells was determined using alamarBlue cytotoxicity assay.

**mSCARB-2 siRNA-mediated Gene Silencing.**

On-TARGET plus SMARTpool mSCARB-2-targeting siRNA (GCAAUAUGAUUAACGGGAC, CAGAUCAACACUUACGUUA, CGGUAGACCAGACGAUCGA, AGGAAGAGCAUGAGUCGUU), as well as non-targeting siRNA (Dharmacon, Thermo Scientific), were diluted with DharmaFECT Cell Culture Reagent (DCCR) (Thermo Scientific) to final concentrations of 5, 10, 25 and 50 nM. The siRNA mixture was then incubated with transfection reagent (Dharmafect-1) at a final volume of 100 µL per well for 30 minutes. The transfection mixture was mixed with NSC-34 cells (10^5^ cells/ 400µL) and seeded onto 24 wells plate. At 48 h.p.t., the transfected cells were harvested for Western blot analysis or subjected to EV71 S41 infection at MOI 10. The culture supernatant was collected for virus titer determination by plaque assay at 48 h.p.i.. Cytotoxicity of mSCARB-2-targeting siRNAs in NSC-34 cells was assessed using alamarBlue cytotoxicity assay.

**Indirect Immunofluorescence Assay (IFA).**

NSC-34 c­ells (1.5×10^5^ cells) or RD cells (10^5^ cells) were seeded on coverslips, infected with EV71 strains at MOI 10 or 1, respectively, for 1 hour and fixed with ice-cold methanol at various time points of post-infection. The cells were then incubated with AF647-conjugated mouse anti-dsRNA antibody (Engscicons, 1:200 dilution), mouse anti-EV71 monoclonal antibody (Merck, MAB979, 1:1,000 dilution), rabbit anti-EV71 antibody (Invitrogen, PA5-32202, 1:1,000 dilution) or rabbit anti-mouse LC3B antibody (Abcam, ab51520, 1:300 dilution), followed by incubation with anti-mouse Alexa Fluor 488 (Millipore, 1 mg/mL, 1:500 dilution) or anti-rabbit Alexa Fluor 647 (Millipore, 1 mg/mL, 1:500 dilution). Cell nuclei were stained with NucBlue® Live ReadyProbes® Reagent (Life Technologies). DABCO (Invitrogen) was used to mount the samples onto glass slides and the edge of the coverslips was subsequently sealed with nail garnish. Isolated exosomes samples were air dried on coverslip prior to fixation and immunostaining. The samples were viewed using conventional optical-fluorescence microscope (Olympus IX81).

**Annexin-V Apoptosis Assay.**

RD and NSC-34 cells were seeded onto 6-well plates at a density of 10^6^ and 10^7^ cells/well and infected with EV71 strains at MOI 1 and 10, respectively. The cell culture supernatants and cells were collected at various time points for Annexin-V and propidium iodide (PI) staining by using Annexin V FITC Apoptosis Detection kit I (BD Pharmingen, USA, #556547). Briefly, the culture medium and cells were collected and resuspended in the binding medium before staining with 2.5 µL of FITC-Annexin V and 5 µL of Propodium Iodide at RT in the dark for 15 minutes. The cell suspension was then analysed on the Sy3200 Cell Sorter (Sony Biotechnology Inc., USA). Mock-infected cells were used as negative control. As a positive control, seeded NSC-34 cells were pre-treated with 1 µM of staurosporine (Sigma) and incubated for 8 hours.

**TUNEL Assay.**

NSC-34 (3×10^4^ cells), SK-N-SH (3×10^4^ cells), SH-SY5Y (3×10^4^ cells) were seeded onto 96-well plates and infected at MOI 1 or 10. At various time points p.i. (36 hours for SK-N-SH and SH-SY5Y cells, 72 hours for NSC-34 cells), the infected cells were fixed with methanol before TUNEL assay was carried out following the manufacturer’s instructions using the TiterTacs Colorimetric Apoptosis Detection Kit (Trevigen). Briefly, cells were permeabilised with cytonin. Endogenous peroxidases were quenched with hydrogen peroxide before Tdt labelling buffer (containing Tdt enzyme and dNTPs conjugated to biotin) was added. The labelling reaction was halted by the addition of a stop buffer. The cells were then incubated with Strep-HRP for 10 minutes, and washed four times before incubation in the dark with TACS Sapphire. The absorbance of the colorimetric products formed was then measured using a microplate reader (Model 680, Bio-Rad) at 450 nm.

**Rapamycin and 3-methyladenine (3MA) Treatment.**

NSC-34 cells (2×10^5^ cells) were seeded overnight on 24-well plates. For autophagy induction assay, the cells were pre-incubated with rapamycin (Sigma-Aldrich) at concentrations of 100, 200 and 500 nM for 3 hours at 37°C. The cells were then washed thrice before infection with S41 at MOI 10. For autophagy inhibition assay, NSC-34 cells were infected with S41 at MOI 10 prior to the addition of 3MA (Sigma-Aldrich) at the concentrations of 1, 2.5, 5 and 10 mM into the culture supernatant. In both assays, the virus culture supernatant was harvested at 48 h.p.i. for viral plaque assay. The cytotoxicity of each drug on NSC-34 cells was assessed using alamarBlue cytotoxicity assay (see section above).

**Exosomes Isolation.**

NSC-34 cells (5×10^6^ cells/T75 flask) were infected with S41, MS and C2 strains at MOI 10. At 48 h.p.i., the viral culture supernatant was harvested and spun down to remove cell debris. The culture supernatant was then incubated with exosome isolation reagent (Life Technologies, 4478359) overnight at 4°C, prior to centrifugation at 10,000 × g for 1 hour. The pellet, which contained the exosomes, was resuspended with PBS for downstream analysis.

**Transmission Electron Microscopy.**

NSC-34 cells (5×10^6^ cells/T75 flask) were infected with S41 at MOI 20. At 24 and 72 h.p.i., the culture supernatant and cells were fixed with PBS buffer containing 2% glutaraldehyde and post-fixed in 1% osmium tetraoxide. The fixed cells were then dehydrated in ascending graded series of ethanol and embedded with Low Viscosity Epoxy Resin (LVER). The samples were sectioned with ultra-microtome (Leica EM UC7) and collected on 200 M nickel grids. The isolated exosomes (section above) was fixed with HEPES buffer containing 1% osmium tetroxide and negatively stained with both phosphor-tungstic acid (PTA) and uranyl acetate (UA). All samples were viewed under transmission electron microscope, JEM-1010 (JEOL, Japan).

**Mouse Model of EV71-infection.**

Two-week-old AG129 (Type I and II IFN-receptors deficient) mice (B&K Universal, UK) were bred and housed in pathogen-free conditions in individual ventilated cages. Infection of mice was carried out via the intraperitoneal (i.p.) route with EV71 strains at a concentration of 1 x 10^7^ PFU/mouse (200 µL in sterile PBS). Uninfected control mice were administered with PBS instead. Mice were weighed and observed daily for a period of twenty days for clinical score and death. Clinical disease and symptoms were graded as follows: 0, healthy; 1, ruffled hair and hunchbacked appearance; 2, limb weakness; 3, paralysis in one limb; 4, paralysis in two limbs; and 5, death. Upon observation of two-limb paralysis, animals were promptly euthanized for ethical reasons.

**Histology of EV71-infected Mice.**

Control and EV71-infected AG129 mice were euthanized at the indicated time points post-infection, or upon observation of one- or two-limb paralysis. Following systemic perfusion with 50 mL sterile PBS and 30 mL of 10% neutral buffered formalin (NBF) (Sigma-Aldrich), the hind limbs, front limbs, spinal cord and brain from the infected mice were harvested and incubated in NBF at RT for 72 hours. Subsequently, bony tissues were decalcified with 10% EDTA (1^st^ Base) for 2 days and washed for one hour with deionized water. Fixed tissues were paraffin embedded, sectioned and stained with hematoxylin and eosin (H&E). Analysis of the sections was performed on blinded samples.

**Determination of Virus Titers in Organs from Infected Mice.**

At days 2, 4 and 6 p.i., EV71-infected AG129 mice were euthanized. Following systemic perfusion with 50mL of sterile PBS, the front and hind limb muscles, spinal cord and brains of infected mice were harvested and weighed before homogenization with a mechanical homogenizer (Omni, USA) in 1 mL of DMEM. The homogenates were clarified at 14,000 r.p.m. for 10 minutes at 4°C. Clarified supernatants were passed through a 0.22 µm syringe-driven filter unit before serial dilution was carried out for plaque assay. Virus titers were expressed as PFU per gram of tissue.

**References**

1. Singh, S., Poh, C.L., & Chow, V.T. Complete sequence analyses of enterovirus 71 strains from fatal and non-fatal cases of the hand, foot and mouth disease outbreak in Singapore (2000). *Microbiol. Immunol.* **46(11),** 801-808 (2002).
2. Brown, B.A. & Pallansch, M.A. Complete nucleotide sequence of enterovirus 71 is distinct from poliovirus. *Virus Res.* **39(2-3),** 195-205 (1995).
3. Wu, Y., et al. The largest outbreak of hand; foot and mouth disease in Singapore in 2008: the role of enterovirus 71 and coxsackievirus A strains. *Int. J. Infect. Dis.* **14(12),** e1076-1081 (2010).
4. Victorio, C.B., Xu, Y., Ng, Q., Chow, V. T. & Chua, K.B. Phenotypic and genotypic characteristics of novel mouse cell line (NIH/3T3)-adapted human enterovirus 71 strains (EV71:TLLm and EV71:TLLmv). *PLoS One* **9(3),** e92719 (2014).
